# Butyrate upregulates endogenous host defense peptides to enhance disease resistance in piglets via histone deacetylase inhibition

**DOI:** 10.1038/srep27070

**Published:** 2016-05-27

**Authors:** Haitao Xiong, Bingxiu Guo, Zhenshun Gan, Deguang Song, Zeqing Lu, Hongbo Yi, Yueming Wu, Yizhen Wang, Huahua Du

**Affiliations:** 1 Key Laboratory of Animal Nutrition and Feed Science (Eastern of China), Ministry of Agriculture, College of Animal Sciences, Zhejiang University, Hangzhou, 310058, P.R. China

## Abstract

Butyrate has been used to treat different inflammatory disease with positive outcomes, the mechanisms by which butyrate exerts its anti-inflammatory effects remain largely undefined. Here we proposed a new mechanism that butyrate manipulate endogenous host defense peptides (HDPs) which contributes to the elimination of *Escherichia coli* O157:H7, and thus affects the alleviation of inflammation. An experiment in piglets treated with butyrate (0.2% of diets) 2 days before *E. coli* O157:H7 challenge was designed to investigate porcine HDP expression, inflammation and *E. coli* O157:H7 load in feces. The mechanisms underlying butyrate-induced HDP gene expression and the antibacterial activity and bacterial clearance of macrophage 3D4/2 cells *in vitro* were examined. Butyrate treatment (*i*) alleviated the clinical symptoms of *E. coli* O157:H7-induced hemolytic uremic syndrome (HUS) and the severity of intestinal inflammation; (*ii*) reduced the *E. coli* O157:H7 load in feces; (*iii*) significantly upregulated multiple, but not all, HDPs *in vitro* and *in vivo* via histone deacetylase (HDAC) inhibition; and (*iv*) enhanced the antibacterial activity and bacterial clearance of 3D4/2 cells. Our findings indicate that butyrate enhances disease resistance, promotes the clearance of *E. coli* O157:H7, and alleviates the clinical symptoms of HUS and inflammation, partially, by affecting HDP expression via HDAC inhibition.

*E. coli* O157:H7, a predominant enterohemorrhagic *Escherichia coli* (EHEC) serotype, can cause acute gastroenteritis that may be complicated by life-threatening systemic sequelae^1^. EHEC is a non-invasive pathogen^2^ that adheres to intestinal cells and forms attaching and effacing lesions^3^. Damage to the intestinal epithelium allows bacterial virulence factors, such as Shiga toxin, to enter the systemic circulation^4^. Bacterial virulence factors circulate and bind to platelets, monocytes, and neutrophils as well as to platelet-monocyte and platelet-neutrophil complexes^5^,^6^. These factors can then be transferred to target organs, including the kidney and the brain^4^. Large amounts of circulating virulence factors can increase the risk of hemolytic uremic syndrome (HUS), in which red blood cells are destroyed and the kidney exhibits glomerular and tubular damage with extensive apoptosis of renal cortical cells^7^,^8^. However, no specific treatment is available for EHEC-induced HUS. Treatment with antibiotics is not recommended because they may increase toxin release and cause complications^9^.

Our previous studies showed that the expression of PR-39, one of host defense peptides (HDPs), was upregulated with ETEC challenge in two breeds of pigs^10^. HDPs are a group of gene encoded, cationic, small peptides that are essential effector molecules of the innate immune system^11^ existing ubiquitously in both plant and animal kingdoms^12^. Defensins and cathelicidins represent the two major classes of HDPs in vertebrates^13^,^14^,^15^. Thus far, 13 β-defensins and 11 cathelicidins have been identified in pigs^12^. The 13 β-defensins are porcine β-defensin 1 (pBD1), pBD2, pBD3, pBD4, pBD104, pBD108, pBD114, pBD123, pBD125, pBD126, pBD129, epididymis protein 2 splicing variant C (pEP2C) and pEP2E^12^,^16^. The 11 porcine cathelicidins are proline-arginine-rich 39-amino-acid peptide (PR-39), proline-phenylalanine-rich prophenin-1 (PF-1) and PF-2, cysteine-rich PG 1 (PG-1) to PG-5, and three porcine myeloid antimicrobial peptides (PMAP)-23, PMAP-36 and PMAP-37^12^. HDPs are produced constitutively by epithelial cells and phagocytes or are induced during inflammation and infection at mucosal surfaces^17^,^18^,^19^. HDPs kill various microorganisms, including Gram-positive and Gram-negative bacteria, viruses, protozoa, and fungi^12^,^20^. HDPs also modulate the immune response by recruiting and promoting elements of the innate immune system^21^,^22^. Because of their antimicrobial and immune-regulatory functions, HDPs have been developed as promising drugs against antibiotic-resistant microbes^20^,^21^,^23^,^24^.

Butyrate, a major species of short-chain fatty acid (SCFA) produced by bacterial fermentation of undigested carbohydrates in the colon^25^, is found to be capable of inducing HDP expression in human, rabbit, chicken, and various enterocytes^16^,^26^,^27^,^28^,^29^. Meanwhile, butyrate plays an important role in intestinalhealth^24^,^29^,^43^ and has been used to treat different inflammatory disease in clinical practice^30^,^31^,^32^,^33^. However, little is known about the mechanism of its anti-inflammatory activity. It is uncertain that if there are connections between the upregulation of HDP expression and the attenuated inflammatory levels after butyrate treatment. Here, an experiment in piglets treated with sodium butyrate (NaB) 2 days before *E. coli* O157:H7 challenge was designed to investigate porcine HDP expression in tissues, inflammation and *E. coli* O157:H7 load in feces. Furthermore, the mechanisms underlying butyrate-induced HDP gene expression were also examined.

## Results

### NaB alleviates clinical symptoms caused by *E. coli* O157:H7 infection

**Figure 1:**
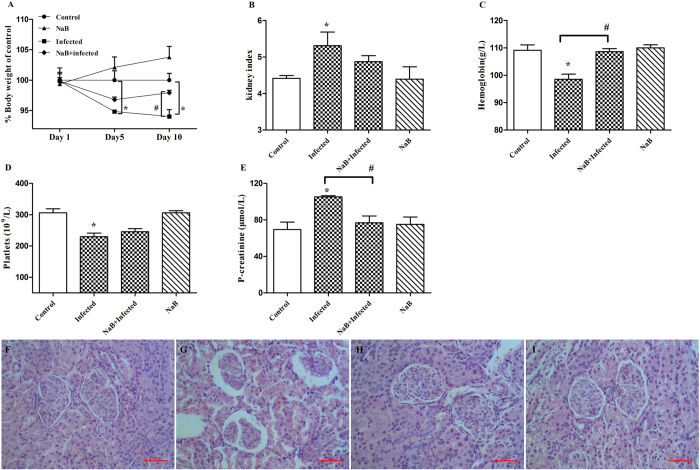
NaB alleviates clinical symptoms caused by *E. coli* O157:H7 inoculation. (**A**) Body weight is shown as a percentage of weight change from days 1 to 10. Pigs developed symptomatic diseases after inoculation with *E. coli* O157:H7. (**B**) Kidney index = kidney weight (g)/body weight (kg). (**C**) Hemoglobin (g/L), (**D**) platelet counts (/L), and (**E**) plasma creatinine levels were analyzed from precaval vein blood samples from the same pigs in (**C**) and (**D**). Data are presented as the mean ± SEM. *Indicates a significant difference (*p* < 0.05) compared to that of control group by One-way ANOVA. ^#^Indicates a significant difference (*p* < 0.05) compared to that of infected group by One-way ANOVA. (**F**) Normal renal cortex from a pig. (**G**) Renal cortex from an *E. coli* O157:H7-challenged pig. (**H**) Renal cortex from a pig fed with NaB and challenged by *E. coli* O157:H7. (**I**) Renal cortex from a pig fed with NaB. The scale bar represents 50 μm.

*E. coli* O157:H7-challenged piglets developed clinical signs of disease, including loss of appetite, ruffled fur, decreased activity and lethargy, after 24 h of infection. However, the piglets treated with NaB showed no symptoms. Body weight loss due to *E. coli* O157:H7 infection in the NaB-treated group occurred before day 5, but animals began to recover by day 10 (Fig. 1A). Compared with the control group, *E. coli* O157:H7-challenged piglets suffered from kidney enlargement (*p* < 0.05, Fig. 1B), anemia (*p* < 0.05, Fig. 1C), thrombopenia (*p* < 0.05, Fig. 1D), and increased plasma creatinine levels (*p* < 0.05, Fig. 1E). In contrast, NaB-treated piglets with *E. coli* O157:H7 infections showed a normal kidney index and normal levels of hemoglobin, platelet counts and plasma creatinine concentrations. The microscopic analysis of piglet kidneys from the *E. coli* O157:H7-challenged group showed that glomeruli were shrunken and tubular epithelial cells were desquamated with typical apoptotic features, such as cell shrinkage and membrane blebbing (Fig. 1G), whereas NaB treatment alleviated these histopathological signs of kidney damage (Fig. 1H). No histopathological signs were observed in either the control group (Fig. 1F) or the NaB group (Fig. 1I).

### NaB reduces *E. coli* O157:H7 counts in feces

**Figure 2:**
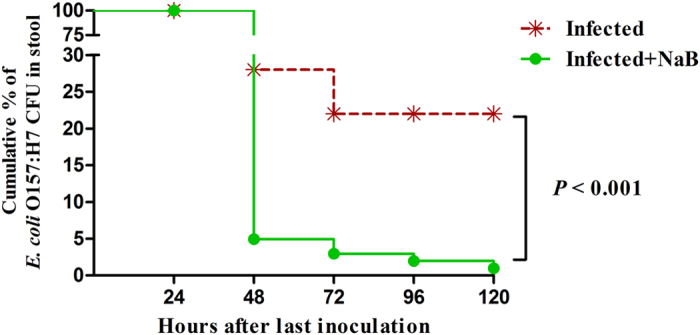
Kaplan-Meier survival plot revealed a reduction of *E. coli* 157:H7 in the stool of infected piglets treated with NaB. There were 6 piglets in each group. ^##^Indicate an extrem significant difference (*p* < 0.01) compared to that of infected group by Log-rank (Mantel-Cox) test.

To investigate whether the positive effects of NaB treatment on clinical symptoms were connected to a reduction of the *E. coli* O157:H7 load in the gut, a Kaplan-Meier survival-plot analysis of colony-forming units (CFUs) in the stool was performed. Compared with the untreated infected piglets, piglets treated with NaB exhibited a greater reduction of *E. coli* O157:H7 shedding in feces (log-rank test, *p* < 0.001). In addition, their *E. coli* O157:H7 counts in the feces decreased by approximately 10-fold (6.08 × 10^7^ vs. 2.46 × 10^6^) at 72 h and by approximately 10^2^-fold (5.09 × 10^7^ vs. 6.28 × 10^4^) at 96 h. The CFU counts of the *E. coli* O157:H7 shed in the feces of infected untreated piglets remained at a high level over time (Fig. 2).

### NaB ameliorates the inflammation caused by *E. coli* O157:H7 infection

**Figure 3:**
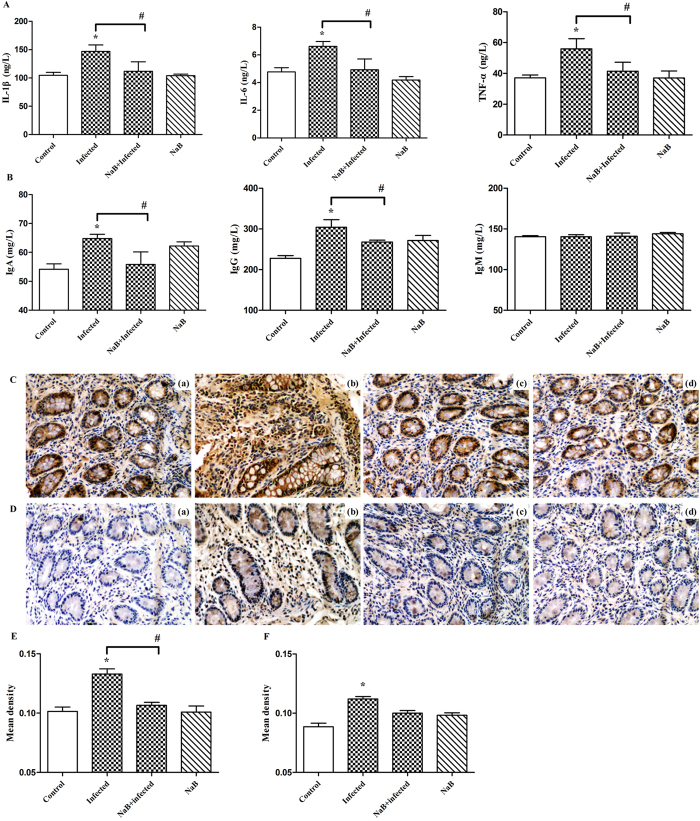
NaB ameliorates the inflammation caused by *E. coli* O157:H7 infection. Concentrations of serum cytokines (**A**) and immunoglobulins (**B**) in NaB− and non-NaB-treated pigs were measured. (**C**) Representative images of the infiltration of CD68^+^ cells. Original magnification ×400. Formalin-fixed, paraffin-embedded 5-μm cross-sections were stained with a primary Ab to CD68^+^. (C**a**) Control, (C**b**) infected, (C**c**) NaB + infected, (C**d**) NaB pigs. (**D**) Representative images of the infiltration of CD177^+^ cells. Original magnification ×400. Formalin-fixed, paraffin-embedded 5-μm cross-sections were stained with a primary Ab to CD177^+^. (D**a**) Control, (D**b**) infected, (D**c**) NaB+infected, (D**d**) NaB pigs. Optical density of the CD68^+^ macrophage infiltrate (**E**) and the CD177^+^ neutrophil infiltrate (**F**). Four to six microscopic fields were randomly selected from each sample. Data plotted represent the mean ± SEM. *Indicates a significant difference (*p* < 0.05) compared to that of control group by One-way ANOVA. ^#^Indicates a significant difference (*p* < 0.05) compared to that of infected group by One-way ANOVA.

Butyrate generally elicits anti-inflammatory effects^34^,^35^,^36^,^37^. To confirm the effects of butyrate on the inflammation caused by *E. coli* O157:H7 infection, the concentrations of pro-inflammatory cytokines and immunoglobulins in serum were measured (Fig. 3A,B). *E. coli* O157:H7 infection significantly increased the levels of pro-inflammatory cytokines (IL-6, IL-1β, and TNF-α) and immunoglobulins (IgA and IgG) compared with the control group. Dietary NaB can prevent *E. coli* O157:H7-induced increases in serum concentrations of IL-6, IL-1β, TNF-α, IgA, and IgG.

The infiltration of CD68^+^ macrophages and CD177^+^ neutrophils into colonic tissue was detected through immunohistochemical analysis (Fig. 3C,D). Macrophages and neutrophils minimally infiltrated the colons of the piglets in the control group and the NaB-treated group. The infiltration of CD68^+^ macrophages and CD177^+^ neutrophils into the colonic lesion area was increased in the *E. coli* O157:H7-challenged group compared with the control group. By contrast, the infiltration of macrophages and neutrophils decreased when the piglets were treated with NaB 2 d before *E. coli* O157:H7 challenge compared with the group challenged with *E. coli* O157:H7 alone.

### NaB stimulates HDP gene expression

**Figure 4:**
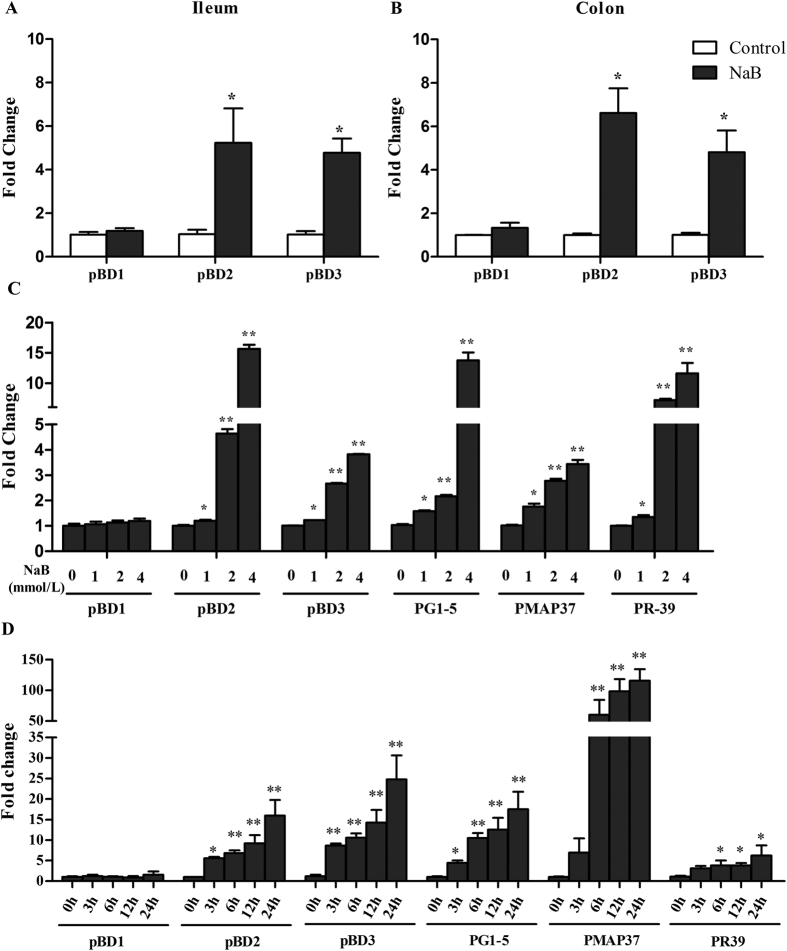
NaB simulates HDP gene expression *in vivo* and *in vitro*. Ileum (**A**) and colon (**B**) tissues of piglets treated with or without NaB (0.2% of diet) for 10 d. Gene expression was analyzed by qPCR. 3D4/2 cells were exposed to the indicated concentrations of NaB for 24 h (**C**) or 4 mmol/L of NaB for 3, 6, 12 or 24 h (**D**) *in vitro*. Data are presented as the mean ± SEM, n = 6. *Indicates a significant difference (*p* < 0.05) and **Indicate an extreme significant difference (*p* < 0.01) compared to that of control group by Students *t*-test.

To further elucidate the effects of NaB on HDP gene expression in piglets, ileum and colon tissues were collected from the control group and the NaB-treated group and subjected to qPCR analysis to examine whether NaB can induce HDP expression *in vivo*. NaB treatment significantly upregulated pBD2 and pBD3 gene expression in the ileum and colon (Fig. 4A,B).

We also stimulated porcine 3D4/2 cell lines with different NaB concentrations for 24 h and with 4 mmol/L NaB for different times. We then performed qPCR analysis. The results show that treatment with NaB for 24 h markedly increased the expression of HDPs, such as pBD2, pBD3, PG1-5 (PG1, PG2, PG3, PG4 and PG5), PMAP37 and PR-39, in 3D4/2 cells in a dose-dependent manner (Fig. 4C). An obvious time-dependent induction of pBD2, pBD3, PG1-5, PMAP37 and PR-39 was also observed (Fig. 4D). These results are consistent with earlier reports on humans and chickens^27^,^28^,^29^. pBD1 gene expression was not changed.

### Butyrate upregulates endogenous HDPs via HDAC inhibition

**Figure 5:**
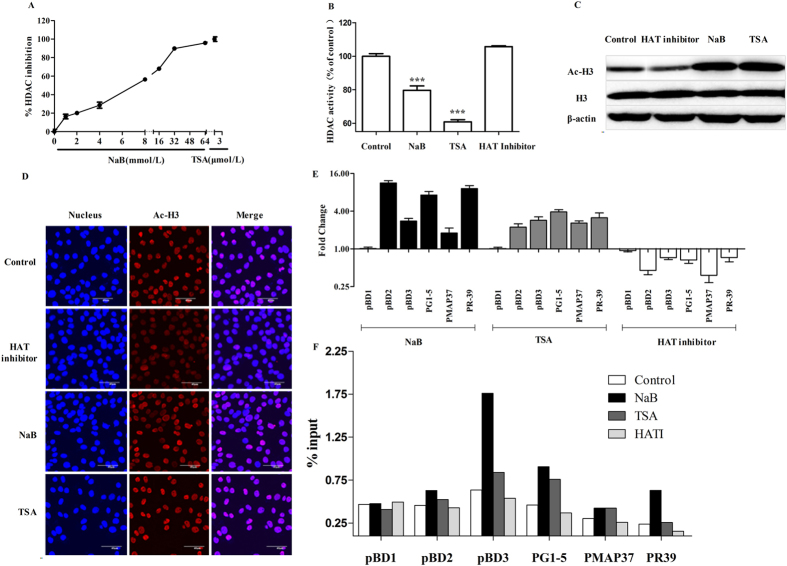
Butyrate upregulates HDP expression via HDAC inhibition. (**A**) Overall inhibition of HDAC enzymes in 3D4/2 cell nuclear extracts by increasing concentrations of butyrate. TSA was used as a reference. (**B**) HDAC activity in 3D4/2 cells was determined. Cells were incubated with NaB, TSA or an HAT inhibitor for 24h. The results were normalized using the control as 100%. ***Indicate an extreme significant difference (*p* < 0.001) compared to that of control group by One-way ANOVA. Western blot (**C**) and immunofluorescence (**D**) analysis of acetylated histone complex H3 in 3D4/2 cells. 3D4/2 cells were incubated with NaB, TSA or HAT inhibitor for 24 h. Western blot analysis of the acetylation of H3 (AcH3) was examined in the nuclear extract. Immunofluorescent staining with anti-AcH3 (red) Ab. Cells were counterstained with DAPI. Effects of butyrate, TSA and HAT inhibitor on HDP gene expression in macrophage 3D4/2 cells. (**E**) 3D4/2 cells were treated with NaB, TSA and an HAT inhibitor for 24 h. cDNA was analyzed by qPCR. Gene expression changes are expressed as the foldchange in transcription in treatedcells with respect to un-treated cells. Numbers <1 denote downregulation, and those >1 indicate upregulation. Data were normalized to GAPDH. (**F**) 3D4/2 cells were treated with NaB, TSA or an HAT inhibitor for 24 h. Samples were analyzed by ChIP using antibodies against H3K9Ac. Purified DNA was analyzed by qPCR using primers specific to the promoters of the indicated genes. Normalized results are shown as a percentage of the input values.

Previous studies in other cell types have demonstrated that butyrate inhibits the activity of HDACs^38^. To determine whether butyrate behaves as an HDAC inhibitor in 3D4/2 cells, we first evaluated whether butyrate could inhibit HDAC enzymes expressed in nuclear cell extracts by incubating nuclear cell extracts with butyrate. Incubation with butyrate showed a significant dose-dependent inhibition of HDAC activity (Fig. 5A). Second, we investigated whether butyrate was able to directly modulate HDAC enzyme activity in 3D4/2 cells by treating cells with NaB, TSA (an HDAC inhibitor) or a histone acetyltransferases (HAT) inhibitor for 24 h. The results show that NaB and TSA could enter 3D4/2 cells to inhibit HDAC activity, whereas the HAT inhibitor had no influence (Fig. 5B). To further demonstrate that butyrate acts as an HDAC inhibitor, we treated 3D4/2 cells with butyrate and quantified the histone H3 acetylation levels by western blot and immunofluorescence. Similar to TSA, butyrate increased histone acetylation, whereas the HAT inhibitor inhibited histone acetylation (Fig. 5C,D). These results suggest that NaB behaves as an HDAC inhibitor in 3D4/2 cells.

HATs and HDACs have opposing activities in regulating gene expression. To verify that HDAC and the HAT inhibitor had opposite effects on HDP gene expression, we treated 3D4/2 cells with NaB, TSA and the HAT inhibitor for 24 h. HDP gene expression was analyzed by qPCR. The results show that both NaB and TSA, HDAC inhibitors, upregulated the expression of 9 HDP genes, including pBD2, pBD3, PG1-5, PMAP37 and PR39, whereas the HAT inhibitor treatment downregulated the expression of these genes (Fig. 5E). Furthermore, ChIP experiments revealed that treatment of 3D4/2 cells with NaB or TSA led to an increase in histone 3 lysine 9 (H3K9Ac) levels at the promoter regions of pBD2, pBD3, PG1-5, PMAP37 and PR39 but not pBD1, whereas the HAT inhibitor inhibited H3K9Ac levels at the promoter regions of these genes (Fig. 5F). H3K9Ac is typically considered a marker of transcriptional activation^39^,^40^. Taken together, these results suggest that butyrate upregulates endogenous HDPs via HDAC inhibition.

### Butyrate augments the antibacterial activity and bacterial clearance of 3D4/2 cells, leading to an amelioration of the inflammation, which is likely due to induction of the endogenous synthesis of HDPs

**Figure 6:**
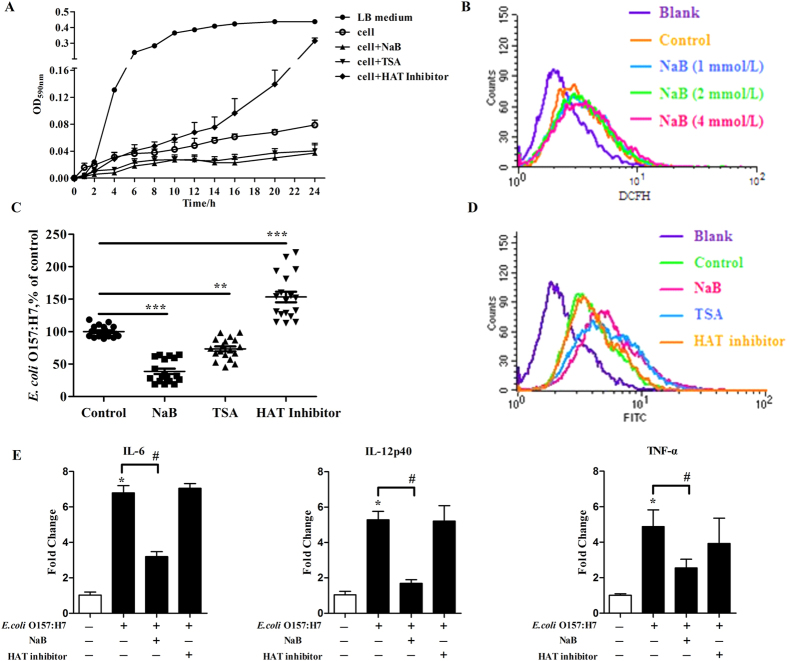
Butyrate enhances antibacterial and bacterial clearance of 3D4/2 cells. (**A**) In the antibacterial assay, 3D4/2 cells were incubated with NaB, TSA or HAT inhibitor for 24 h. The cells were lysed, and the supernatants were incubated with *E. coli* O157:H7. Bacterial turbidity was measured at OD_590 nm_ using a microplate reader. (**B**) In the oxidative burst assay, 3D4/2 cells were incubated with the indicated concentration of NaB for 24 h, washed by PBS, and then incubated for 1 h with 2′,7′-dichlorodihydrofluorescein diacetate (DCFH). The fluorescein was analyzed by FACS. In the phagocytosis assay, 3D4/2 cells were treated with NaB, TSA oran HAT inhibitor for 24 h and then infected with *E. coli* O157:H7 for 1 h. After the adherent and non-adherent bacteria were killed by gentamycin, the cells were washed, lysed, and placed onto LB agar and the colonies were counted (**C**). In another experiment, treated 3D4/2 cells were co-incubated with FITC-dextran for 2 h. The percentage of cellular uptake of FITC-dextran was analyzed by FACS (**D**). The results were normalized using the control as 100%. **Indicates a significant difference (p < 0.01) and ***Indiate an extreme significant difference (*p* < 0.001) compared to that of control group by One-way ANOVA. (**E**) 3D4/2 cells were treated with NaB and HAT inhibitor for 24 h and then infected with *E. coli* O157:H7 for 1 h.cDNA was analyzed by qPCR. Data were normalized to GAPDH. Data are representative of two independent experiments. Error bars represent mean ± SEM.

The intracellular environment of defense cells, such as macrophages, is hostile to bacteria because of the secretion of antimicrobial peptides and the generation of reactive oxygen intermediates. To investigate whether the antibacterial activity of 3D4/2 cells was enhanced by NaB-induced HDP gene expression, we stimulated 3D4/2 cells with NaB, TSA and HAT inhibitor for 24 h, lysed the cells, incubated the cell lysates with *E. coli* O157:H7, and measured bacterial turbidity every 2 h. The results show that bacteria grew well in the LB medium. NaB-treated 3D4/2 lysates, which exhibited NaB-induced HDP expression, significantly suppressed bacterial growth, whereas HAT inhibitor-treated cell lysates, which showed a downregulation of HDPs, had a weaker ability to kill bacteria compared with the lysates from untreated cells (Fig. 6A). To further rule out the possibility that butyrate’s augmentation of antibacterial activity was not attributable to a change in the oxidative burst activity of 3D4/2 cells, we treated 3D4/2 cells with different concentrations of butyrate for 24 h and then incubated them with DCFH. The distribution of the fluorescein was analyzed by FACS. The results show that butyrate has no effect on the oxidative burst in macrophage cells (Fig. 6B). These results indicate that butyrate enhances the antibacterial activity of macrophages through the upregulation of HDP expression.

We further examined the influence of butyrate on *E. coli* O157:H7 phagocytosis by 3D4/2 cells. 3D4/2 cells were treated with NaB, TSA and the HAT inhibitor for 24 h and then infected with *E. coli* O157:H7 for 1 h. After the adherent and non-adherent bacteria were killed by gentamycin, cells were washed, lysed, and placed onto LB agar and the resulting colonies were counted. The results show that the number of living *E. coli* O157:H7 phagocytosed by macrophages treated with NaB and TSA was significantly less than that of the control group, whereas the number of living *E. coli* O157:H7 phagocytosed by macrophages treated with the HAT inhibitor was significantly greater than that of the control (Fig. 6C). Two possible explanations may account for these results: NaB suppressed bacterial phagocytosis by macrophages or NaB enhanced bacterial clearance by macrophages. We evaluated the effect of NaB on the phagocytic capacity of 3D4/2 macrophages by FACS analysis. The incubation of 3D4/2 cells with NaB, TSA and the HAT inhibitor for 24 h had no effect on macrophage phagocytic capacity (Fig. 6D). Previous reports have shown that HDPs, such as LL-37 and HNP1-3, enhance bacterial clearance by phagocytes^41^,^42^,^43^. By upregulating HDP gene expression, NaB enhanced the bacterial clearance of 3D4/2 cells, whereas the HAT inhibitor suppressed bacterial clearance by downregulating HDP gene expression. These results indicate that butyrate enhances the bacterial clearance of macrophages through the upregulation of HDP expression.

Furthermore, we tested whether butyrate could regulate macrophage function by a butyrate-induced augmentation of the antibacterial activity and bacterial clearance. 3D4/2 cells were treated with NaB and the HAT inhibitor for 24 h and then infected with *E. coli* O157:H7 for 1 h. Proinflammatory mediator mRNA levels were examined by qPCR. As shown in Fig. 6E, compared with the control, *E. coli* O157:H7 infection strongly increased the transcript levels of proinflammatory cytokines IL-6, TNF-α and IL-12p40. NaB treatment decreased the expression of these proinflammatory mediator genes, whereas the HAT inhibitor treatment had no effect on the expression of these genes when compared with cells infected with *E. coli* O157:H7 alone. These results indicate that butyrate has anti-inflammatory effects on macrophages.

Collectively, these results suggest that butyrate treatment augments antibacterial activity and bacterial clearance, leading to amelioration of inflammation, and that these effects are likely due to the induction of endogenous synthesis of HDPs.

## Discussion

We have demonstrated, in an experimental piglet model of *E. coli* O157:H7 infection, that oral administration of sodium butyrate led to a substantially improved clinical course and outcome (Fig. 1), a significant decrease of the *E. coli* O157:H7 load in feces (Fig. 2) and alleviation of inflammation in the intestine (Fig. 3). Our data support the hypothesis that the mechanism of this therapeutic effect of sodium butyrate operates, in part, through its effect on endogenous HDP expression (Fig. 4). We also described the mechanism by which sodium butyrate modulates HDP expression via the inhibition of HDAC (Fig. 5).

Earlier reports have shown that butyrate-producing bacteria (*C. butyricum*) reduce *E. coli* O157:H7 counts in feces. Although the mechanism remains elusive, the proposed explanation is that butyrate production induces bactericidal effects *in vivo*^44^ and that butyrate can inhibit bacterial growth at high doses^45^. Similarly, our results showed that high butyrate concentrations (256 or 512 mmol/L) were required to kill or inhibit bacterial growth *in vitro* (see Supplementary Fig. S1 online). Because the majority of butyrate can be quickly absorbed in the upper digestive intestine after butyrate is orally administered^46^,^47^,^48^,^49^,^50^, the butyrate concentration required to inhibit bacteria in the colon is difficult to achieve. The mechanism by which bacterial load is reduced is unlikely achieved through the direct bactericidal activity of butyrate. In the current study, we proposed a new mechanism in which butyrate has a regulatory effect on endogenous HDP expression, which contributes to the elimination of pathogens and leads to the alleviation of inflammation. Here, we showed that the oral intake of sodium butyrate by *E. coli* O157:H7-infected piglets led to a reduced clinical severity, a reduced bacterial fecal load, and an amelioration of intestinal inflammation along with an upregulation of endogenous HDP expression. HDPs are critical to the barrier defense provided by the innate immune system, and deficits in HDPs production can increase susceptibility to infections^51^. The production of high levels of HDP mRNA reflects a strategy employed by the host to intensify the innate defense for protection against the invading pathogens. Treatment with butyrate increases the transcription of HDPs, which enhances disease resistance in piglets.

We next investigated the mechanisms underlying butyrate-induced HDP gene expression. It is widely known that butyrate inhibits HDAC activity in many cell types. We investigated whether butyrate exerts its HDP-induced effects by acting as an HDAC inhibitor. In the present study, we showed that butyrate phenocopies a well-characterized HDAC inhibitor, TSA, in macrophages. Like TSA, butyrate inhibited HDAC enzymes in 3D4/2 cells and nuclear cell extracts (Fig. 5A,B). Both butyrate and TSA increased the overall levels of histone H3 acetylation, whereas the HAT inhibitor inhibited the overall levels of histone H3 acetylation (Fig. 5C,D), suggesting that butyrate acts as an HDAC inhibitor in 3D4/2 cells.

In addition, we found that butyrate and TSA, HDAC inhibitors, and an HAT inhibitor have opposing activities to regulate HDPs gene expression. Both butyrate and TSA upregulate pBD2, pBD3, PG1-5, PMAP37 and PR39 gene expression, whereas the HAT inhibitor downregulates the expression of these genes (Fig. 5E). Furthermore, we performed ChIP experiments and demonstrated that treatment of 3D4/2 cells with butyrate or TSA led to an increase in H3K9Ac levels at the promoter regions of pBD2, pBD3, PG1-5, PMAP37 and PR39, whereas the HAT inhibitor decreased H3K9Ac levels at the promoter regions of these genes (Fig. 5F). H3K9Ac is typically considered a marker of ongoing transcription^39^,^40^. Collectively, these data strongly support the hypothesis that butyrate upregulates HDP gene expression via HDAC inhibition.

Finally, we tested whether butyrate has ameliorative effects on macrophages in a model of inflammation induced by *E. coli* O157:H7 infection. It is noteworthy that the HDAC inhibitor, butyrate, upregulated HDP gene expression and led to an enhancement of antibacterial activity and bacterial clearance by macrophages. This change resulted in a reduction in the number of living *E. coli* O157:H7 cells and an amelioration of the inflammatory response, which is consist with *in vivo* results. In contrast, the HAT inhibitor inhibited HDP gene expression and led to the suppression of antibacterial activity and bacterial clearance by macrophages (Fig. 6). Together, these data suggest that butyrate augments the antibacterial activity and bacterial clearance of 3D4/2 cells, leading to an amelioration of inflammation, which is due to the induction of the endogenous synthesis of HDPs.

In this study, we use piglets as a model rather than mice of rats because of that there are more morphological and physiological similarities, such as renal and digestive systems, between human and porcine than for rodent. This study focused on *E. coli* O157:H7 infections because of the clinical relevance and no specific treatment available^9^. New strategies, such as toxin-component vaccines, toxin neutralizers, and monoclonal antibodies that inhibit toxic effects, have been investigated to prevent or treat diseases caused by *E. coli* O157:H7^52^,^53^,^54^,^55^. The antimicrobial peptide cathelicidin LL-37 also shows a protective effect against *E. coli* O157:H7 infection^51^. Our results demonstrate a novel strategy that uses butyrate to stimulate endogenous HDP production and prevent or treat this type of infection.

In conclusion, we demonstrated that butyrate, a natural product of digestion, can be administered to induce endogenous HDP expression, which, in turn, enhances disease resistance, promotes the clearance of *E. coli* O157:H7, and alleviates clinical symptoms and inflammation, and we also elucidated the mechanism of butyrate-induced HDP expression is via the inhibition of HDACs. Our results may also provide a novel method by which dietary substances, such as SCFAs, can potentially be used to manipulate the expression of endogenous HDPs and to boost barrier defenses of innate immunity and disease resistance. Butyrate can also be developed as a new drug candidate to treat *E. coli* O157-mediated disease.

## Materials and Methods

### Reagents

Sodium butyrate (purity above 99%) was purchased from Sigma-Aldrich (Shanghai, China).Vancomycin, streptomycin, gentamycin and cefixime tellurite sorbitol MacConkey agar were purchased from Aladdin (Shanghai, China). ELISA kits for TNF-α, IL-1β, IL-6, IgA, IgG, and IgM were purchased from Biovol Bio-Technology Co., Ltd. (Shanghai, China). A SYBR Premix Ex *Taq*™ kit was purchased from Takara (Dalian, China). The antibodies for acetylated histone H3 (rabbit polyclonal) and histone H3 (rabbit polyclonal) and the H3K9Ac were purchased from Santa Cruz Biotechnology (Burlingame, CA, USA). The Amplite™ Fluorometric HDAC Activity Assay Kit and TSA were purchased from AAT Bioquest^®^, Inc. (Sunnyvale, CA, USA). The HAT-inhibitor Epigenetic Multiple Ligand was purchased from Merck (Darmstadt, Germany). The BCA Protein Assay Kit, Chromatin Immunoprecipitation (ChIP) Assay Kit and DNA purification columns were purchased from Beyotime (Shanghai, China).

### Bacterial strains and cultures

*E. coli* (O157:H7 ATCC 43889 strain) was grown overnight at 37 °C in LB broth and harvested by centrifugation. The pellet was washed twice with sterile PBS and resuspended in a solution containing 10% (w/v) NaHCO_3_ and 20% (w/v) sucrose in sterile PBS. The final bacterial concentration was adjusted to 10^9^ CFU/mL^51^.

### Piglet model of infection

Animal protocols were approved by the animal care committee of Zhejiang University in accordance with the Guide for the Care and Use of Agricultural Animals in Research and Teaching. The methods were carried out in “accordance” with the approved guidelines.

A total of 24 weaned piglets (Duroc × Landrace × Yorkshire) were treated with vancomycin (1 g/kg of diets) and streptomycin (5 g/kg of diets) for 3 d to reduce the normal levels of commensal flora. Then, all piglets were weighed and randomly grouped into four treatments with 6 piglets per group. Piglets were fed with a standard antibiotic-free diet mixed, with or without 0.2% sodium butyrate, throughout the trial period. After two days, the piglets were orally infected with 100 mL of the prepared bacterial suspension at a dose equivalent to 10^11^ CFU/pig every other 24 h for 3 d. Control animals received 100 mL of sterile solution containing 10% (w/v) NaHCO_3_ and 20% (w/v) sucrose. Feces were collected at 24, 48, 72, 96 and 120 h after the last bacterial inoculation was administered.

### Specimen collection

At the end of the experiment, all piglets were narcotized and slaughtered. Tissues from the ileum and colon were collected and immediately frozen in liquid nitrogen. Blood samples were stored in coagulating tubes and centrifuged at 1000 × *g* at 4 °C for 30 min to obtain the serum. The samples were stored at −80 °C until further analysis. Hemoglobin, platelet, and creatinine levels in the blood obtained from the precaval vein were analyzed using an automatic biochemical analyzer.

### Bacterial counts in feces

Feces were weighed, homogenized, suspended in sterile PBS, and serially diluted in PBS. Dilutions were inoculated on sorbitol MacConkey agar (SMAC) plates containing cefixime (0.5 mg/L) and potassium tellurite (2.5 mg/L). After an overnight incubation at 37 °C, the selected sorbitol-negative colonies (white colonies) were counted and evaluated to determine *E. coli* O157:H7 content using multiplex PCR assays^56^.

### Immunohistochemical analysis of macrophages and neutrophils in colonic tissue

The middle section of the colon was removed for immunohistochemical analysis as described previously^57^. Four to six microscopic fields were randomly selected from each animal, and the images were analyzed using Image-Pro Plus 6 (Media Cybernetics, Rockville, MD, USA).

### Measurement of cytokines and immunoglobulins in the serum

The concentrations of the cytokines TNF-α, IL-1β, and IL-6, as well as those of the immunoglobulins IgA, IgG, and IgM, in the serum were measured using commercially available porcine ELISA kits according to the manufacturer’s instructions.

### Quantitative real-time PCR analysis of porcine gene expression

Total RNA was isolated with TRIzol reagent. cDNA was synthesized using a reverse transcription kit. PCR was performed using a SYBR Premix Ex *Taq*™ kit on a Step One Plus^TM^ real-time PCR System (Applied Biosystems, Carlsbad, CA, USA). The gene-specific primers are presented in Table 1. mRNA expression levels were determined using the 2^−ΔΔ*Ct*^ method^58^ with porcine GAPDH as a reference.

### Antibacterial activity of 3D4/2 cells

3D4/2 macrophages in 12-well cell culture plates were treated with or without NaB, trichostatin A (TSA) and the HAT inhibitor Epigenetic Multiple Ligand^59^ for 24 h. The cells were washed with PBS twice, lysed with 0.1% Triton X-100, and centrifuged at 8000 × *g* for 15 min at 4 °C. Cell suspensions of 2 × 10^4^ CFU *E. coli* O157:H7 in 20% LB medium containing 1 mmol/L NaH_2_PO_4_ and 25 mmol/L NaHCO_3_ were added to a 96-well culture plate. The plate was incubated at 37 °C. The bacterial turbidity was measured every 2 h at OD_590 nm_ using a microplate reader. In the oxidative burst assay experiment, 3D4/2 cells in 12-well cell culture plates were washed with PBS and treated with NaB for 24 h. The cells were washed with PBS and then incubated with 10 μmol/L of 2′, 7′-dichlorodihydrofluorescein diacetate (DCFH) for 1 h. The DCFH fluorescence distribution of 10,000 cells was detected by flowcytometric (FACS) analysis at an excitation wavelength of 488 nm and an emission wavelength of 535 nm.

### Phagocytosis assay of 3D4/2 cells

3D4/2 cells in 12-well cell culture plates were washed with PBS and treated with or without NaB (4 mmol/L), TSA (3 μmol/L) and HAT inhibitor (50 μmol/L) for 24 h. The cells were then infected with *E. coli* O157:H7 (MOI = 10) for 1 h. After the free and adherent bacteria were killed by 50 μg/mL gentamycin and washed with PBS, the cells were lysed with 0.1% Triton X-100 in PBS. The lysates were placed on LB agar, and the colonies were counted. In other experiments, treated 3D4/2 cells were co-incubated with fluorescein isothiocyanate (FITC)-dextran for 2 h in an incubator at 37 °C in the dark. The percentage of cellular uptake of FITC-dextran was analyzed by FACS.

### HDAC activity detection in 3D4/2 cells

The HDAC activity of 3D4/2 cells was detected with an Amplite™ Fluorometric HDAC Activity Assay Kit according to manufacturer’s protocol. The nuclear extract was diluted to a final concentration of 5 μg/well and incubated with increasing concentrations of NaB (0–64 mmol/L). A well-characterized HDAC inhibitor, TSA, was used as a positive control. In other experiments, 3D4/2 cells were seeded in a 6-well cell culture plate and treated with or without NaB (4 mmol/L), TSA (3 μmol/L) and HAT inhibitor (50 μmol/L) for 24 h. Then, cells were collected and HDAC activity in nuclear extracts was determined using a fluorescent assay kit.

### Western blot analysis

The concentration of protein in extracts from 3D4/2 cells were determined using a BCA Protein Assay Kit. Extracts containing equal quantities of proteins (50 μg) were resolved on polyacrylamide gels and transferred to PVDF membranes. The membranes were blocked for nonspecific binding for 30 min (5% skimmed protein in PBS) and incubated overnight at 4 °C with antibodies for acetylated histone H3 (Ac-H3) and histone H3 (H3). Blots were developed using ECL detection reagents, exposed on Kodak Xdmat blue XB-1 film, and quantified by ImageJ software.

### Immunofluorescence analysis of acetylated histone H3

3D4/2 cells were seeded in glass-bottomed dishes (MatTek Corporation, Ashland, USA) and treated with NaB (4 mmol/L), TSA (3 μmol/L) and HAT inhibitor (50 μmol/L) for 24 h, washed with PBS and fixed in 100 μL 4% paraformaldehyde for 15 min, and washed with PBS three times for 3 min. The cells were permeabilized in 100 μL 0.5% Triton X-100 for 20 min at room temperature, washed with PBS three times for 3 min, and blocked in 100 μL 1% BSA for 30 min. The cells were incubated in 100 μL antibodies for acetylated histone H3 (Ac-H3) (1:200) in 1% BSA at 4 °C in a humidified container overnight. The cells were washed three times for 5 min with PBST prior to incubation in 100 μL Cy3-conjugated goat anti-rabbit IgG in 1% BSA for 60 min at ambient temperature in the dark. The cells were washed with PBST and then mounted with DAPI for 5 min, after which they were washed with PBS, and the fluorescence was detected using a confocal microscope.

### Chromatin immunoprecipitation (ChIP) assay

ChIP was performed according to the protocol described previously^60^. Briefly, 3D4/2 cells were treated with or without NaB (4 mmol/L), TSA (3 μmol/L), and HAT inhibitor (50 μmol/L) for 24 h. The cells were then cross-linked with formaldehyde and sonicated. The resulting cell lysates were immunoprecipitated with 2 μg of acetylated histone H3 (Lys 9) antibody or the IgG control. The precipitated protein-DNA complexes were subjected to proteinase treatment. The recovered DNA was purified and analyzed by qPCR using gene-specific primers (Table 2).

### Statistics

Data are represented as the mean ± SEM. The difference between groups was determined by Student’s *t*-test or, if there were more than two groups, by ANOVA. *E. coli* O157:H7 CFU in stool was compared between the treatment groups by Kaplan-Meier survival plot and was determined by log-rank (Mantel-Cox) test. All of the data are presented as mean ± SEM, and a *p*-value at *p* < 0.05 was taken as statistically significant.

## Additional Information

**How to cite this article**: Xiong, H. *et al.* Butyrate upregulates endogenous host defense peptides to enhance disease resistance in piglets via histone deacetylase inhibition. *Sci. Rep.* **6**, 27070; doi: 10.1038/srep27070 (2016).

## Supplementary Material

Supplementary Information

## Figures and Tables

**Table 1:** Primer sequences for qPCR analysis.

Gene	Forward primer	Reverse primer
pBD1	TCCTTGTATTCCTCCTCA	ACACGCCTTTATTCCTTA
pBD2	ACCTGCTTACGGGTCTTG	CTCTGCTGTGGCTTCTGG
pBD3	GAAGTCTACAGAAGCCAAAT	GGTAACAAATAGCACCATAA
PG1-5	TCTCTTGGTCACAGGTTCA	GACACAGACGCAGAACCTA
PMAP37	GCTGTGTGACTTCAAGGAGAA	GAAATCTCCTGACACCCTCATT
PR-39	CAAGGCCACCTCCGTTTT	CCACTCCATCACCGTTTTCC
IL-6	TGGCTACTGCCTTCCCTACC	CAGAGATTTTGCCGAGGATG
TNF-α	CCAATGGCAGAGTGGGTATG	TGAAGAGGACCTGGGAGTAG
IL-12p40	GGCCAGTACACCTGTCGCA	TTGGGCTCTTTCTGGTCTT
GAPDH	ACTCACTCTTCCACTTTTGATGCT	TGTTGCTGTAGCCAAATTCA

**Table 2:** Primer sequences for ChIP analysis.

Gene	Forward primer	Reverse primer
pBD1	GGCCCTTGAGGATGTGATAAA	CTGTGGGCATGTCACTTAGAT
pBD2	TTACCCACAGAAGCTGGTAATG	AGGAAGGAAGGAAGGAAGGA
pBD3	CCCAATGTTCATAGCAGCTTTATT	GTAGTAGCCCATTGTGTCATGTA
PG1-5	CCCACTGCCCAGCAATC	TGCGGGACCCTCCTTTAT
PMAP37	ACTCAGTAAGCGTGGGTATTG	GGCTTCACAGTCCAGGTATT
PR-39	CTTCCCAGTAGAGGCATGTTATT	GCCACAGTTTGAGGTGATTTG

## References

[b1] Mayr U. B. *et al.* Rectal single dose immunization of mice with Escherichia coli O157:H7 bacterial ghosts induces efficient humoral and cellular immune responses and protects against the lethal heterologous challenge. Microb Biotechnol 5, 283–294, 10.1111/j.1751-7915.2011.00316.x (2012). 22103353 PMC3815788

[b2] Kaper J. B., Nataro J. P. & Mobley H. L. Pathogenic Escherichia coli. Nature reviews. Microbiology 2, 123–140, 10.1038/nrmicro818 (2004). 15040260

[b3] Jerse A. E., Yu J., Tall B. D. & Kaper J. B. A Genetic-Locus of Enteropathogenic Escherichia-Coli Necessary for the Production of Attaching and Effacing Lesions on Tissue-Culture Cells. P Natl Acad Sci USA 87, 7839–7843, 10.1073/pnas.87.20.7839 (1990). PMC54845 2172966

[b4] Karpman D., Sartz L. & Johnson S. Pathophysiology of typical hemolytic uremic syndrome. Seminars in thrombosis and hemostasis 36, 575–585, 10.1055/s-0030-1262879 (2010). 20865634

[b5] Stahl A. L., Sartz L., Nelsson A., Bekassy Z. D. & Karpman D. Shiga toxin and lipopolysaccharide induce platelet-leukocyte aggregates and tissue factor release, a thrombotic mechanism in hemolytic uremic syndrome. PloS one 4, e6990, 10.1371/journal.pone.0006990 (2009). 19750223 PMC2735777

[b6] Tazzari P. L. *et al.* Flow cytometry detection of Shiga toxins in the blood from children with hemolytic uremic syndrome. Cytometry. Part B, Clinical cytometry 61, 40–44, 10.1002/cyto.b.20022 (2004). 15351981

[b7] Karpman D. *et al.* Apoptosis of renal cortical cells in the hemolytic-uremic syndrome: *in vivo* and *in vitro* studies. Infection and immunity 66, 636–644 (1998). 9453620 10.1128/iai.66.2.636-644.1998 PMC107951

[b8] Kaneko K. *et al.* Apoptosis of renal tubular cells in Shiga-toxin-mediated hemolytic uremic syndrome. Nephron 87, 182–185, 45909 (2001). 11244315 10.1159/000045909

[b9] Wong C. S., Jelacic S., Habeeb R. L., Watkins S. L. & Tarr P. I. The risk of the hemolytic-uremic syndrome after antibiotic treatment of Escherichia coli O157 : H7 infections. New Engl J Med 342, 1930–1936, 10.1056/Nejm200006293422601 (2000). 10874060 PMC3659814

[b10] Gao Y. H. *et al.* Expression pattern of porcine antimicrobial peptide PR-39 and its induction by enterotoxigenic Escherichia coli (ETEC) F4ac. Vet Immunol Immunop 160, 260–265, 10.1016/j.vetimm.2014.05.012 (2014). 24929581

[b11] Zasloff M. Antimicrobial peptides of multicellular organisms. Nature 415, 389–395, 10.1038/415389a (2002). 11807545

[b12] Sang Y. M. & Blecha F. Porcine host defense peptides: Expanding repertoire and functions. Dev Comp Immunol 33, 334–343, 10.1016/j.dci.2008.05.006 (2009). 18579204

[b13] Ganz T. Defensins: Antimicrobial peptides of innate immunity. Nature Reviews Immunology 3, 710–720, 10.1038/Nri1180 (2003). 12949495

[b14] Lehrer R. I. Primate defensins. Nature Reviews Microbiology 2, 727–738, 10.1038/Nrmicro976 (2004). 15372083

[b15] Bevins C. L. & Salzman N. H. Paneth cells, antimicrobial peptides and maintenance of intestinal homeostasis. Nature Reviews Microbiology 9, 356–368 (2011). 21423246 10.1038/nrmicro2546

[b16] Zeng X. F. *et al.* Induction of Porcine Host Defense Peptide Gene Expression by Short-Chain Fatty Acids and Their Analogs. PloS one 8, ARTN e72922.10.1371/journal.pone . 0072922 (2013). 10.1371/journal.pone.0072922 PMC3758276 24023657

[b17] Zhang G. L. *et al.* Molecular cloning and tissue expression of porcine beta-defensin-1. FEBS letters 424, 37–40, 10.1016/S0014-5793(98)00134-3 (1998). 9537511

[b18] Sang Y. M., Patil A. A., Zhang G. L., Ross C. R. & Blecha F. Bioinformatic and expression analysis of novel porcine beta-defensins. Mamm Genome 17, 332–339, 10.1007/s00335-005-0158-0 (2006). 16596454

[b19] Islam D. *et al.* Downregulation of bactericidal peptides in enteric infections: A novel immune escape mechanism with bacterial DNA as a potential regulator. Nat Med 7, 180–185, 10.1038/84627 (2001). 11175848

[b20] Marr A. K., Gooderham W. J. & Hancock R. E. W. Antibacterial peptides for therapeutic use: obstacles and realistic outlook. Curr Opin Pharmacol 6, 468–472, 10.1016/j.coph.2006.04.006 (2006). 16890021

[b21] Hancock R. E. W. & Sahl H. G. Antimicrobial and host-defense peptides as new anti-infective therapeutic strategies. Nat Biotechnol 24, 1551–1557, 10.1038/Nbt1267 (2006). 17160061

[b22] Hilchie A. L., Wuerth K. & Hancock R. E. Immune modulation by multifaceted cationic host defense (antimicrobial) peptides. Nature chemical biology 9, 761–768, 10.1038/nchembio.1393 (2013). 24231617

[b23] Ashby M., Petkova A. & Hilpert K. Cationic antimicrobial peptides as potential new therapeutic agents in neonates and children: a review. Curr Opin Infect Dis 27, 258–267, 10.1097/Qco.0000000000000066 (2014). 24722240

[b24] Di Luca M., Maccari G. & Nifosi R. Treatment of microbial biofilms in the post- antibiotic era: prophylactic and therapeutic use of antimicrobial peptides and their design by bioinformatics tools. Pathog Dis 70, 257–270, 10.1111/2049-632x.12151 (2014). 24515391

[b25] Canani R. B. *et al.* Potential beneficial effects of butyrate in intestinal and extraintestinal diseases. World journal of gastroenterology : WJG 17, 1519–1528, 10.3748/wjg.v17.i12. 1519 (2011). 21472114 PMC3070119

[b26] Raqib R. *et al.* Improved outcome in shigellosis associated with butyrate induction of an endogenous peptide antibiotic. Proc Natl Acad Sci USA 103, 9178–9183, 10.1073/pnas.0602888103 (2006). 16740661 PMC1482586

[b27] Schauber J. *et al.* Histone-deacetylase inhibitors induce the cathelicidin LL-37 in gastrointestinal cells. Mol Immunol 41, 847–854, 10.1016/j.molimm.2004.05.005 (2004). 15261456

[b28] Schauber J. *et al.* Expression of the cathelicidin LL-37 is modulated by short chain fatty acids in colonocytes: relevance of signalling pathways. Gut 52, 735–741, 10.1136/Gut.52.5.735 (2003). 12692061 PMC1773650

[b29] Sunkara L. T. *et al.* Butyrate Enhances Disease Resistance of Chickens by Inducing Antimicrobial Host Defense Peptide Gene Expression. PloS one 6, ARTN e27225. 10.1371/journal.pone. 0027225 (2011). PMC3208584 22073293

[b30] Scheppach W. & Weiler F. The butyrate story: old wine in new bottles? Curr Opin Clin Nutr. 7, 563–567, 10.1097/00075197-200409000-00009 (2004). 15295277

[b31] Vernia P. *et al.* Topical butyrate improves efficacy of 5-ASA in refractory distal ulcerative colitis: results of a multicentre trial. Eur J Clin Invest 33, 244–248, 10.1046/j.1365-2362.2003.01130.x (2003). 12641543

[b32] Scheppach W. *et al.* Effect of Butyrate Enemas on the Colonic Mucosa in Distal Ulcerative-Colitis. Gastroenterology 103, 51–56 (1992). 1612357 10.1016/0016-5085(92)91094-k

[b33] Luhrs H. *et al.* Butyrate inhibits NF-kappaB activation in lamina propria macrophages of patients with ulcerative colitis. Scand J Gastroenterol 37, 458–466 (2002). 11989838 10.1080/003655202317316105

[b34] Schwab M. *et al.* Role of nuclear hormone receptors in butyrate-mediated up-regulation of the antimicrobial peptide cathelicidin in epithelial colorectal cells. Mol Immunol 44, 2107–2114, 10.1016/j.molimm.2006.09.016 (2007). 17055059

[b35] Klampfer L., Huang J., Sasazuki T., Shirasawa S. & Augenlicht L. Inhibition of interferon gamma signaling by the short chain fatty acid butyrate. Mol Cancer Res 1, 855–862 (2003). 14517348

[b36] Meijer K., de Vos P. & Priebe M. G. Butyrate and other short-chain fatty acids as modulators of immunity: what relevance for health? Curr Opin Clin Nutr 13, 715–721, 10.1097/Mco.0b013e32833eebe5 (2010). 20823773

[b37] Sina C. *et al.* G Protein-Coupled Receptor 43 Is Essential for Neutrophil Recruitment during Intestinal Inflammation. Journal of immunology 183, 7514–7522, 10.4049/jimmunol.0900063 (2009). 19917676

[b38] Davie J. R. Inhibition of histone deacetylase activity by butyrate. The Journal of nutrition 133, 2485S–2493S (2003). 12840228 10.1093/jn/133.7.2485S

[b39] Karmodiya K., Krebs A. R., Oulad-Abdelghani M., Kimura H. & Tora L. H3K9 and H3K14 acetylation co-occur at many gene regulatory elements, while H3K14ac marks a subset of inactive inducible promoters in mouse embryonic stem cells. Bmc Genomics 13, Artn 424. 10.1186/1471-2164- 13–424 (2012). 22920947 PMC3473242

[b40] Musselman C. A. *et al.* Binding of the CHD4 PHD2 finger to histone H3 is modulated by covalent modifications. Biochem J 423, 179–187, 10.1042/Bj20090870 (2009). 19624289 PMC2885444

[b41] Wan M. *et al.* Antimicrobial peptide LL-37 promotes bacterial phagocytosis by human macrophages. J Leukocyte Biol. 95, 971–981, 10.1189/Jlb.0513304 (2014). 24550523

[b42] Wan M., Sabirsh A., Wetterholm A., Agerberth B. & Haeggstrom J. Z. Leukotriene B-4 triggers release of the cathelicidin LL-37 from human neutrophils: novel lipid-peptide interactions in innate immune responses. Faseb J. 21, 2897–2905, 10.1096/fj.06-7974com (2007). 17446260

[b43] Soehnlein O. *et al.* Neutrophil primary granule proteins HBP and HNP1-3 boost bacterial phagocytosis by human and murine macrophages. J Clin Invest 118, 3491–3502, 10.1172/Jci35740 (2008). 18787642 PMC2532980

[b44] Takahashi M. *et al.* The effect of probiotic treatment with Clostridium butyricum on enterohemorrhagic Escherichia coli O157 : H7 infection in mice. Fems Immunol Med Mic. 41, 219–226, 10.1016/j.femsim.2004.03.010 (2004). 15196571

[b45] Nakanishi N. *et al.* Regulation of virulence by butyrate sensing in enterohaemorrhagic Escherichia coli. Microbiol-Sgm. 155, 521–530, 10.1099/Mic.0.023499-0 (2009). 19202100

[b46] Sarker P. *et al.* Phenylbutyrate Counteracts Shigella Mediated Downregulation of Cathelicidin in Rabbit Lung and Intestinal Epithelia: A Potential Therapeutic Strategy. PloS one 6, ARTN e20637.10.1371/journal.pone. 0020637 (2011). 10.1371/journal.pone.0020637 PMC3108617 21673991

[b47] Egorin M. J., Yuan Z. M., Sentz D. L., Plaisance K. & Eiseman J. L. Plasma pharmacokinetics of butyrate after intravenous administration of sodium butyrate or oral administration of tributyrin or sodium butyrate to mice and rats. Cancer Chemoth Pharm 43, 445–453, 10.1007/s002800050922 (1999). 10321503

[b48] Roda A. *et al.* A new oral formulation for the release of sodium butyrate in the ileo-cecal region and colon. World J Gastroentero 13, 1079–1084 (2007). 10.3748/wjg.v13.i7.1079 PMC4146871 17373743

[b49] Manzanilla E. G. *et al.* Effects of butyrate, avilamycin, and a plant extract combination on the intestinal equilibrium of early-weaned pigs. J Anim Sci 84, 2743–2751, 10.2527/Jas.2005-509 (2006). 16971576

[b50] Guilloteau P. *et al.* From the gut to the peripheral tissues: the multiple effects of butyrate. Nutrition research reviews 23, 366–384, 10.1017/S0954422410000247 (2010). 20937167

[b51] Chromek M., Arvidsson I. & Karpman D. The Antimicrobial Peptide Cathelicidin Protects Mice from Escherichia coli O157:H7-Mediated Disease. PloS one 7, ARTN e46476.10.1371/journal.pone. 0046476 (2012). 10.1371/journal.pone.0046476 PMC3471911 23077510

[b52] Bitzan M. Treatment options for HUS secondary to Escherichia coli O157:H7. Kidney international. Supplement, S62–66, 10.1038/ki.2008.624 (2009). 19180140

[b53] Mukhopadhyay S. & Linstedt A. D. Manganese blocks intracellular trafficking of Shiga toxin and protects against Shiga toxicosis. Science 335, 332–335, 10.1126/science.1215930 (2012). 22267811 PMC5367627

[b54] Dean-Nystrom E. A., Gansheroff L. J., Mills M., Moon H. W. & O’Brien A. D. Vaccination of pregnant dams with intimin(O157) protects suckling piglets from Escherichia coli O157:H7 infection. Infection and immunity 70, 2414–2418 (2002). 11953378 10.1128/IAI.70.5.2414-2418.2002 PMC127944

[b55] Smith D. R. *et al.* A two-dose regimen of a vaccine against type III secreted proteins reduced Escherichia coli O157:H7 colonization of the terminal rectum in beef cattle in commercial feedlots. Foodborne pathogens and disease 6, 155–161, 10.1089/fpd.2008.0136 (2009). 19105625

[b56] Gannon V. P. J. *et al.* Use of the flagellar H7 gene as a target in multiplex PCR assays and improved specificity in identification of enterohemorrhagic Escherichia coli strains. J Clin Microbiol 35, 656–662 (1997). 9041407 10.1128/jcm.35.3.656-662.1997 PMC229645

[b57] Toyoda T. *et al.* Gene expression analysis of a Helicobacter pylori-infected and high-salt diet-treated mouse gastric tumor model: identification of CD177 as a novel prognostic factor in patients with gastric cancer. Bmc Gastroenterol 13, Artn 122.10.1186/1471-230x- 13–122 (2013). 23899160 10.1186/1471-230X-13-122 PMC3734037

[b58] Schmittgen T. D. & Livak K. J. Analyzing real-time PCR data by the comparative C-T method. Nat Protoc 3, 1101–1108, 10.1038/nprot.2008.73 (2008). 18546601

[b59] Mai A. *et al.* Epigenetic multiple ligands: Mixed Histone/Protein methyltransferase, acetyltransferase, and class III deacetylase (Sirtuin) inhibitors. J Med Chem 51, 2279–2290, 10.1021/jm701595q (2008). 18348515

[b60] Shi Y. H. *et al.* Specific immunotherapy in combination with Clostridium butyricum inhibits allergic inflammation in the mouse intestine. Sci Rep-Uk 5, Artn 17651. 10.1038/Srep17651 (2015). PMC4667269 26627845

