# Supplementary material for: Butyrate upregulates endogenous host defense peptides to enhance disease resistance in piglets via histone deacetylase inhibition
Source: Sci Rep. 2016 May 27;6:27070. doi: 10.1038/srep27070 (PMC4882515; doi:10.1038/srep27070)
Supplement: Supplementary Information [file srep27070-s1.doc]

**Online Supporting Material**

**Butyrate upregulates endogenous host defense peptides to enhance disease resistance in piglets via histone deacetylase inhibition**

Haitao Xiong, Bingxiu Guo, Zhenshun Gan, Deguang Song, Zeqing Lu, Hongbo Yi, Yueming Wu , Yizhen Wang^*^, Huahua Du^*^

Supplementary Figure S1. Effect of NaB on *E. coli* O157:H7 growth *in vitro*. *E. coli* O157:H7 incubated with different concentrations of NaB in LB medium containing 1 mmol/L NaH_2_PO_4_ and 25 mmol/L NaHCO_3_ at 37 °C in an incubator. Bacterial density at OD_590 nm_ was measured at 12 h as an indication of bacterial growth. Data plotted represent the mean value ± SEM, ** indicates p<0.01.
